# Use of Irradiated Polymers after Their Lifetime Period

**DOI:** 10.3390/polym10060641

**Published:** 2018-06-09

**Authors:** David Manas, Miroslav Manas, Ales Mizera, Jan Navratil, Martin Ovsik, Katarina Tomanova, Stanislav Sehnalek

**Affiliations:** 1 Faculty of Applied Informatics, Tomas Bata University in Zlin, CEBIA-Tech, Nad Stranemi 4511, 760 05 Zlin, Czech Republic; dmanas@utb.cz (D.M.); manas@utb.cz (M.M.); sehnalek@utb.cz (S.S.); 2 Faculty of Technology, Tomas Bata University in Zlin, Vavreckova 275, 760 01 Zlin, Czech Republic; ovsik@utb.cz; 3 SKODA AUTO a.s., tr. Vaclava Klementa 869, 293 01 Mlada Boleslav-Mlada Boleslav II, Czech Republic; jan.navratil@skoda-auto.cz; 4 Department of Polymer Processing, Slovak University of Technology in Bratislava, Vazovova 5, 812 43 Bratislava, Slovakia; katarina_tomanova@stuba.sk

**Keywords:** polymer recycling, radiation cross-linking, end-of-life cycle, polymers, mechanical properties

## Abstract

This article deals with the study of the utilisation of irradiated HDPE products after their end-of-life cycle. Today, polymer waste processing is a matter of evermore intensive discussion. Common thermoplastic waste recycling—especially in the case of wastes with a defined composition—is generally well-known—and frequently used. On the contrary, processing cross-linked plastics is impossible to do in the same way as with virgin thermoplastics—mainly due to the impossibility of remelting them. The possibility of using waste in the form of grit or a powder, made from cross-linked High Density PolyEthylene (rHDPEx) products, after their end-of-life cycle, as a filler for virgin Low Density PolyEthylene (LDPE) was tested in a matrix. It was found that both the mechanical behaviour and processability of new composites with an LDPE matrix, with rHDPEx as a filler, depend—to a high degree—on the amount of the filler. The composite can be processed up to 60% of the filler content. The Polymer Mixture Fluidity dropped significantly, in line with the amount of filler, while the mechanical properties, on the other hand, predominantly grew with the increasing amount of rHDPEx.

## 1. Introduction

The plastics industry plays a very important role in the global economy. In the last few years, polymers have become one of the most important materials. Polymers are used in a wide range of applications—e.g., packaging, household applications, sport and leisure time accessories. Plastic materials play a very important role in the design of electrical and electronic products, medical applications and automotive, aircraft and aerospace industry components. In co-dependence with the growth of the plastics industry, the amount of generated waste of both types of plastic parts in the end-of-life cycle and post industrial waste phases is also increasing. Research works concerning real time prediction of some types of polyolefin or biodegradable polymer films were also carried out [1,2]. The quality of the waste differs from the point-of-view of its origin. Waste generated during the production process—e.g., runners and sprues from injection moulding—or off-specification products from injection moulding, extrusion, blow moulding, thermoforming and other processes are easy to identify and are usually of high quality. This declaration is correct for plastic products made from one type of polymers. Multicomponent plastic parts, or composites, mainly come from injection moulding or extrusion processes. The quality of the polymers derived from end-of-life products depends on the way the waste is collected, and could lead to problems. This differs from country to country. End-of-life products can be collected separately or together with other waste [3]. For these reasons, this waste could lead to mixed plastics of unknown composition and could also be potentially contaminated by organic or inorganic fractions [4]. This makes the waste processing/recycling process much more complicated. Today, there is a huge amount of plastic waste to dispose of, and only about 30% of this is effectively recycled, while roughly the same amount is landfilled. The rest is used for energy recovery [5].

The reduction of plastic waste and the discovery of effective ways to recycle plastics is the greatest challenge for scientists, producers and users of polymer parts in the future. Today, the most common plastic waste processing method is mechanical recycling, which includes various steps leading to the conversion of the waste into a useful material. These steps cover the collection, sorting, washing/cleaning, and grinding of the waste. The crushed material can be used directly as an addition to a virgin polymer and could be re-granulated in the next possible step [5,6,7,8,9,10,11]. These steps may occur in different order, multiple times, or not at all, depending on the origins and composition of the waste. In the simplest case, it will be in the form of runners and sprues from injection-moulding or waste from its beginning to its production by extrusion, where the origin and type of waste is clear, and this material is usually clean. In the case of mixed plastics, the process is much more complex and covers additional steps—e.g., sorting the waste according to the type and colour of the polymers, and steps leading to organic and inorganic contaminants being removed [4]. Various principles and methods are used for the sorting step including Fourier-Transform Near-InfraRed spectroscopy (FT-NIR), Optical Colour Recognition, Electrostatic Separation, Flotation, Magnetic Separation, X-ray Detection, or manual sorting [12,13,14,15]. During the re-granulation of recycled materials process, melting filtration is used to remove non-melting components—e.g., wood, paper, rubber particles [16], or polymers with higher melting temperatures [17]. Mechanical/material recycling represents a way of processing waste into new products without any changes to the macromolecular structure. Usually, this type of new product has very good properties, possibly even approaching those of the original item. Usually, the reground material/waste is mixed with virgin resin. Another method is chemical recycling, which refers to the decomposition of macromolecular structures in order to generate low molecular-weight components such as monomers for their utilisation in polymeric materials, synthetic gases, mixtures of liquids for the petrochemical industry, or as fuels and so on. The chemical recycling process includes many techniques—including chemolysis, pyrolysis, fluid catalic cracking, hydrogen technologies, or gasification [18,19,20]. Material recycling with the remelting of a new material obtained from a waste is suitable for the thermoplastics industry. The remelting process is not applicable for polymers modified by irradiation cross-linking. Because of the ever-growing knowledge of polymer property changes after irradiation, the new technology of radiation cross-linking is applied in the production of a huge number of products [21,22,23].

Over the last few decades, the industrial use of radiation cross-linking for standard as well as engineering polymers has increased rapidly. The cross-linking process enables one to change the properties of plastics, and especially, to improve their mechanical, chemical and thermal properties [23,24,25,26]. Using the irradiation cross-linking technology, it is possible to prepare plastics with properties that accord to consumer requirements, i.e., so-called “tailored plastics”. Some plastics, e.g., polyethylene (PE), can be cross-linked without any need of cross-linking agents. Other polymers, polyamides for instance, require the addition of additives prior to processing to enable the cross-linking reaction during the radiation process [21,22]. Linked with the increasing production of cross-linked plastic products, the production of waste rapidly increases. Up until today, there still is no efficient technology of cross-linked plastic waste processing to its disposal. Despite improvement in their properties, radiation cross-linked thermoplastic materials lose their natural ability of being re-melted repeatedly, due to the formation of networks [25,27,28,29,30,31,32].

This article describes one of the possible ways of processing recycled cross-linked High Density Polyethylene (rHDPEx), originally used for the production of underfloor heating systems. There is a huge amount of waste from this production to dispose of, and millions of kilometres of heating pipes are installed in buildings. It is therefore necessary to think about what will be done with this material/processing waste or waste at the end of such productts’ lifetimes.

## 2. Materials and Methods

Crushed cross-linked rHDPEx from underfloor heating pipes as the filler added to the LDPE matrix was used in this study. Granulate of virgin LDPE as a matrix, and recycled irradiated rHDPEx, were used for the preparation of two types of mixtures.

### 2.1. Material

The wide availability of rHDPEx was the main reason for the use of this polymer in the research presented herein. This material was supplied in the form of heating pipes used for underfloor heating systems. The pipes were crushed using the low-speed knife mill, type MASKIN AB RAPIS S-33010, screen size 5 mm and ground using the toothed disc mill, type Condux. To obtain crushed or powdered material for further processing, the particles with dimension higher than 1 mm were separated from the powder using screen 1 mm. The pipes were originally irradiated by beta (electron) radiation, with an energy of 10 MeV, and irradiation dose of 165 kGy. Low Density PolyEthylene (LDPE—780E, Dow Chemical Company, Midland, MI, USA) was chosen as the polymer matrix regarding their ease of processability, low price and wide availability. LDPE granulate was used as the polymer matrix and rHDPEx in grit or powder states were used as the filler.

### 2.2. Irradiation

Irradiation of used HDPE pipes originally determined for use in underfloor heating systems was originally carried out at the premises of BGS Beta-Gama-Servise Company (Wiehl, Germany), Germany. Electron beam radiation, with the dose of 165 kGy, and energy of 10 MeV, was used for their irradiation. The cross-linking of used HDPE was realized without the use of a cross-linking agent. Gel Content Measurement and a Dosimeter were used to determine and find proof of correctness. The correct radiation dose was checked by a Nylon FTN 60-00 dosimeter; and the analysis using a Genessis (Goleta, CA, USA) 5 spectrophotometer in correspondence with the ASTM 51261 standard [33]. Gel content was measured according to the ASTM D7567 standard [34], which is determined by means of solvent extraction with xylene. The dose of 165 kGy corresponds to 60% gel content.

### 2.3. Specimen Preparation

**Figure 1:**
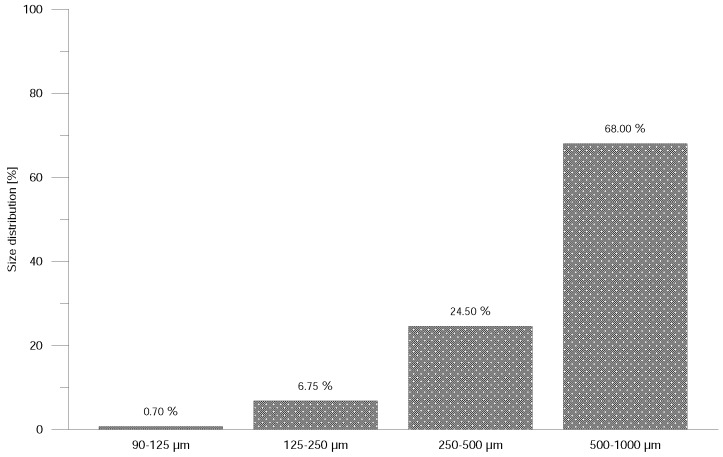
Powder size distribution.

**Figure 2:**
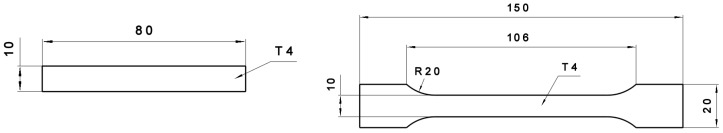
Dimensions of testing specimen: type 1 (**left**) and type 1A (**right**).

From crushed and powdered material, two types of mixtures, which differ from each other by filler state (grit or powder, rHDPEx), were prepared: Mixture A (LDPE granulate + rHDPEx grit) and Mixture B (LDPE granulate + rHDPEx powder). Crushed (particle size of 1–5 mm) or powdered rHDPEx was mixed together with virgin LDPE granulate in a laboratory fluid mixer. A size analysis was performed using Retsch AS 200 Basic equipment (Figure 1) for determination of particle size distribution. Figure 1 shows that dimensions of 68% particles are in interval 0.5–1.0 mm and only less than 0.05% particles are smaller than 0.09 mm. From this mixture, in concentrations ranging 10–60 wt. % of rHDPEx in virgin LDPE, specimens according to the ISO 527 standard [35] were prepared by injection moulding using an Arburg Allrounder 470H injection moulding machine type 1, specimens for Impact test and type 1A, specimens for Tensile test. Dimensions of specimens are in Figure 2 and the processing conditions are given in Table 1.

Ten samples were prepared for each testing method and statistical evaluation was realized in programmes TestXpert II (V3.31, Zwick/Roell, Ulm, Germany), MS Excel 2016 (Microsoft, Redmond, WA, USA) and MiniTab (v16, State, College, PA, USA). In all figures, arithmetic mean and standard deviation are used.

### 2.4. Melt Flow Measurement

A Type LMI 4003Dynisco Kayeness Melt-flow Indexer was used to measure the Melt Flow Index (MFI). The measurements were performed according to ISO 1133 standard [36], and a load of 2.16 kg at the temperature of 190 ${}^{\circ }$C.

### 2.5. Polymer Mixture Fluidity

**Figure 3:**
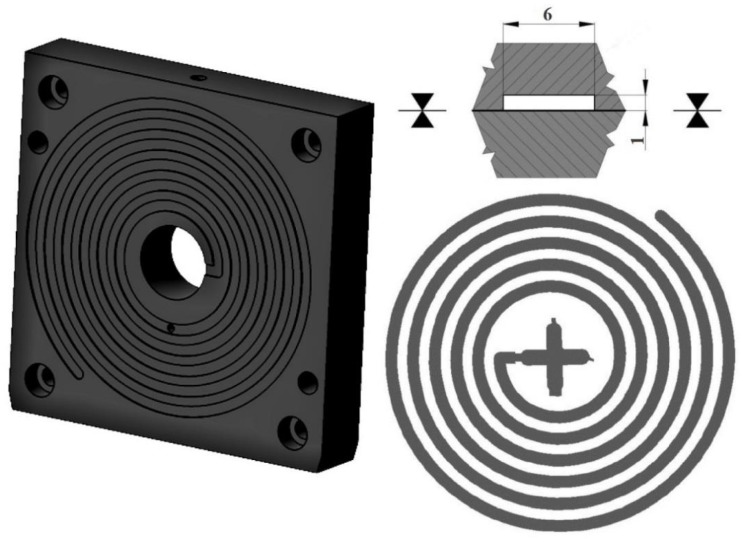
Spiral cavity mould.

A Modified Spiral Test was performed using a spiral cavity mould [37], with a spiral length of 2000 mm; the shape of the cavity is rectangular, with the dimensions of 6 mm × 1 mm (Figure 3). Arburg Allrounder 470H injection moulding machine (Loßburg, Germany) was used for testing purposes.

### 2.6. Tensile Test

A ZWICK 1456 tensile machine (Ulm, Germany) was used for the estimation of tensile behaviour. Measurements were carried out according to the ISO 527 standard [35] at ambient (23 ${}^{\circ }$C) and elevated (80 ${}^{\circ }$C) temperatures with crosshead speed of 50 mm/min. The E-modulus, ultimate tensile strength and strain were all evaluated from these measurements. Conditioning was taken for five days at temperature of 23 ${}^{\circ }$C and relative humidity of 50%.

### 2.7. Shore D Hardness (ShD)

An OMAG AFFRI ART 13 Shore D hardness tester (Varese, Italy) was used for hardness testing in accordance with the ISO 868 standard [38], therefore holding time was 15 s.

### 2.8. Micro Indentation

Micro-indentation tests were performed using a CSM Instruments Micro Combi tester (Needham, MA, USA), according to the ISO 14577 standard [39]. A Vickers type indentor with a diamond tip was used, with a maximal load of 1 N; loading and unloading rate 2 N/min; and holding time 90 s.

### 2.9. Impact Test

The Charpy impact test was carried out on Zwick HIT50P equipment (Ulm, Germany) at an ambient temperature of 23 ${}^{\circ }$C according to the ISO 179 standard [40]. In this impact hammer test, 50 J of potential energy was used for research purposes. Fifteen samples were tested (Figure 2), and their impact toughness values were evaluated in the TestXpert II (ZWICK, Ulm, Germany), MS Excel (Redmond, Washington, USA) and MiniTab 16 (Minitab Ltd., Brandon Court Unit, Coventry, UNITED KINGDOM) programmes. Arithmetic mean and standard deviation were used as the statistical parameters in this measurement process. Conditioning was taken for five days at temperature of 23 ${}^{\circ }$C and relative humidity of 50%.

### 2.10. Structural Analysis

A LEICA RM2255 microtome (Eisfeld, Germany) was used for the preparation of samples (thickness of 35 μm) and an OLYMPUS BX41 microscope (Tokyo, Japan) was used for the structural analysis. Equally, the structure was investigated on the fracture area with nitrogen-cooled bodies using a JEOL 7500F scanning electron microscope (Tokyo, Japan).

## 3. Results and Discussion

**Figure 4:**
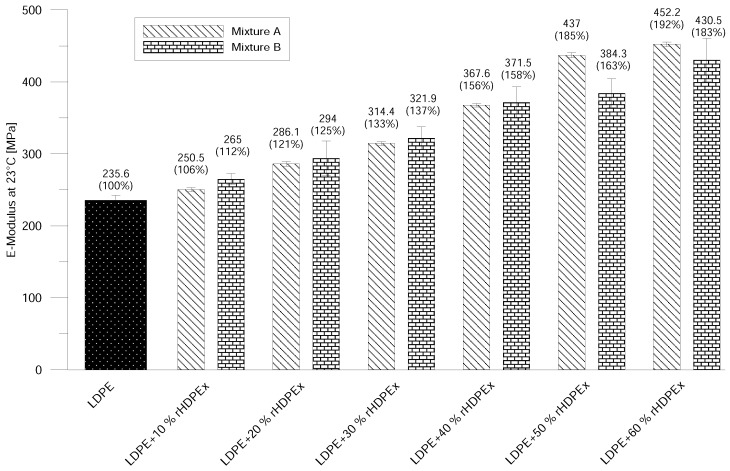
E-modulus at ambient temperature—Mixtures A and B.

**Figure 5:**
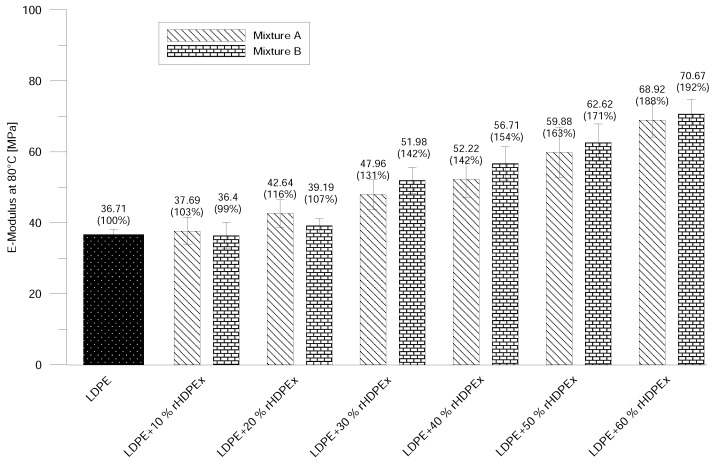
E-modulus at elevated temperature—Mixtures A and B.

Tensile testing was chosen because it provides the most complex overview of the mechanical properties of a tested material. For better comparison of the measured data, the values of virgin LDPE are also shown in the figures. The Elastic Modulus of Mixture A at room temperature already begins to show improvements by the addition of only 10% of rHDPEx. Adding more filler causes a dramatic hike—up to 452 MPa at the highest concentration (60%)—which represents a nearly double higher elastic modulus in comparison with virgin LDPE. It is possible to observe the same tendency in the elastic modulus results of Mixture B (Figure 4). Differences in the elastic modulus values—as measured for both mixtures, are insignificant and lie within the measurement error limits. Roughly the same increase (i.e., double) of the elastic modulus was found by tests at the elevated temperature of 80 ${}^{\circ }$C (Figure 5), at the highest concentration point of the filler.

**Figure 6:**
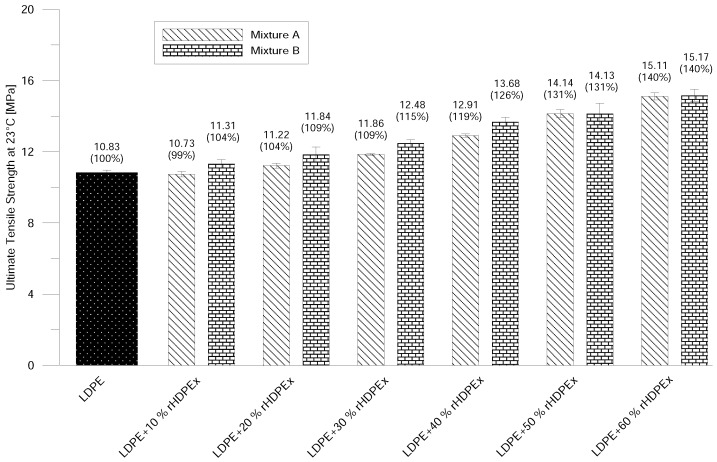
Ultimate tensile strength at ambient temperature—Mixtures A and B.

**Figure 7:**
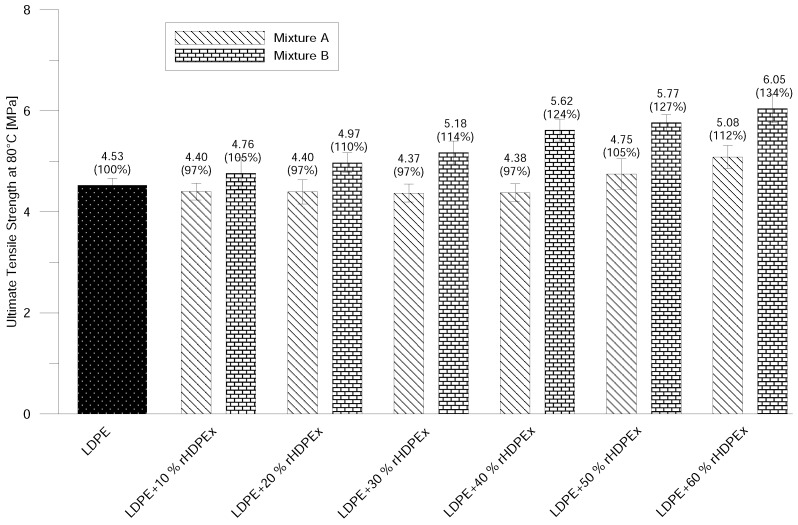
Ultimate tensile strength at elevated temperature—Mixtures A and B.

Ultimate Tensile Strength increases continually with the amount of filler content and, at the concentration of 60% of rHDPEx in the LDPE matrix, the increase is about 40% (Figure 6). That correlates very well with the tendency of the increase of the elastic modulus at ambient conditions. No significant differences in the comparison of Mixtures A and B were found. The differences of the measured values are within the error measurement range. At elevated temperatures, the ultimate tensile strength in Mixture A—due to the addition of 10–40% of filler—decreases slightly, and, at the maximum filler content (60%), the ultimate tensile strength is 12% higher than that of the virgin LDPE in the case of Mixture A. With regard to the measurement error, composite Mixture A—containing a 10–50% filler content—has the same Ultimate Tensile Strength as the LDPE matrix. A slight increase is recorded at a dose of 60 wt. % fillers. Mixture B shows a gradual improvement in tensile strength, and, at the highest rHDPEx concentration, it is 34% higher than that of LDPE (Figure 7).

**Figure 8:**
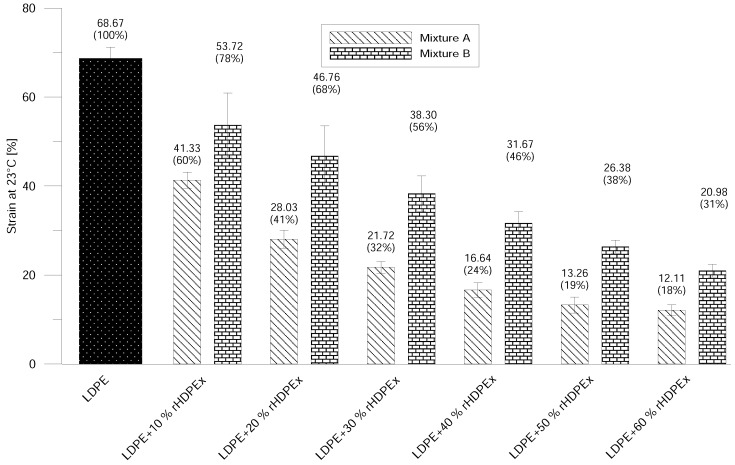
Strain at ambient temperature—Mixtures A and B.

**Figure 9:**
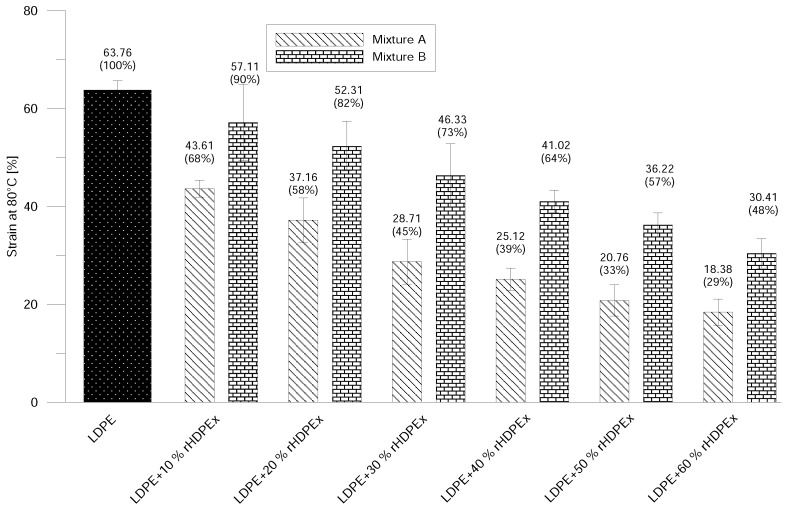
Strain at elevated temperature—Mixtures A and B.

A dramatic drop in strain at break was observed—even at the lowest filling concentration (Figure 8). The addition of 10% rHDPEx as a filler resulted in a significant decrease of strain to 60% Mixture A, and higher concentrations of filler in the LDPE matrix caused gradual decreases in the strain value. The difference at the highest concentration of the filler represents a decrease of strain of about 82% (Mixture A) or 69% (Mixture B). However, the drop in strain values for B is significantly slower in comparison to Mixture A. It is possible to observe the same tendency by means of strain measurement at the elevated (80 ${}^{\circ }$C) temperature (Figure 9). Just as with normal temperature test results, a more pronounced decrease in Strains in Mixture A, as compared to Mixture B, can be observed, even at elevated temperatures. This corresponds very well with the increase of E-modulus and tensile strength—and attests to the influence of rHDPEx as a filler, both from the point of view of filler content and particles size.

**Figure 10:**
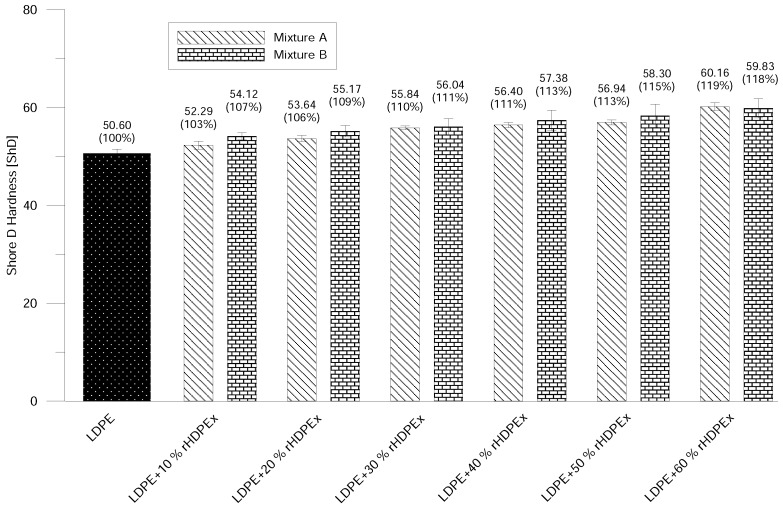
Shore D hardness—Mixtures A and B.

**Figure 11:**
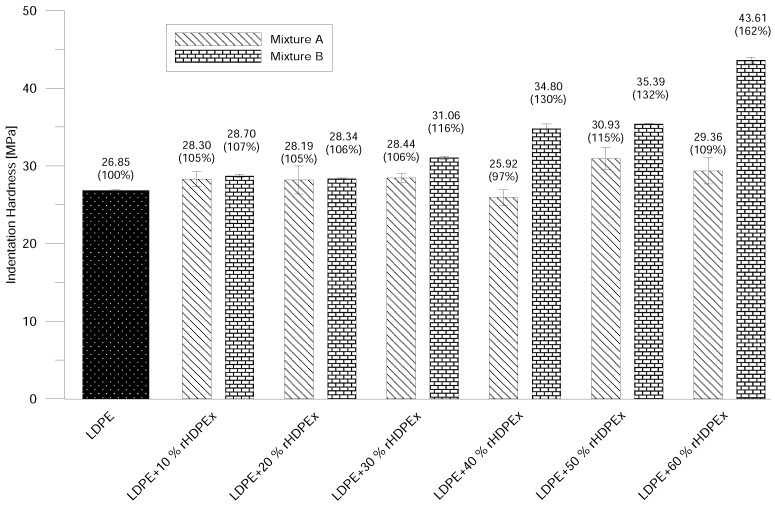
Micro hardness testing—Mixtures A and B.

Shore D Hardness is in line with the increase of rHDPEx in the LDPE matrix. The ShD hardness continually rises to nearly 20% above the hardness of virgin LDPE with the highest filler content. There is only an insignificant difference in hardness when comparing Mixtures A and B (Figure 10); the measured values mainly lay within the measurement error interval. The micro-hardness of the tested material—represented by the indentation hardness $H_{IT}$—shows only very low changes with the rising level of the filler/grit concentration in the case of Mixture A. On the other hand, increasing content of the rHDPEx filler in the form of powder (Mixture B) causes significant changes in the indentation hardness—especially for filler batches of 30–60 wt. %. At the concentration level of 60 wt. % of rHDPEx powder, the indentation hardness is 62% higher—as compared to virgin LDPE (Figure 11).

**Figure 12:**
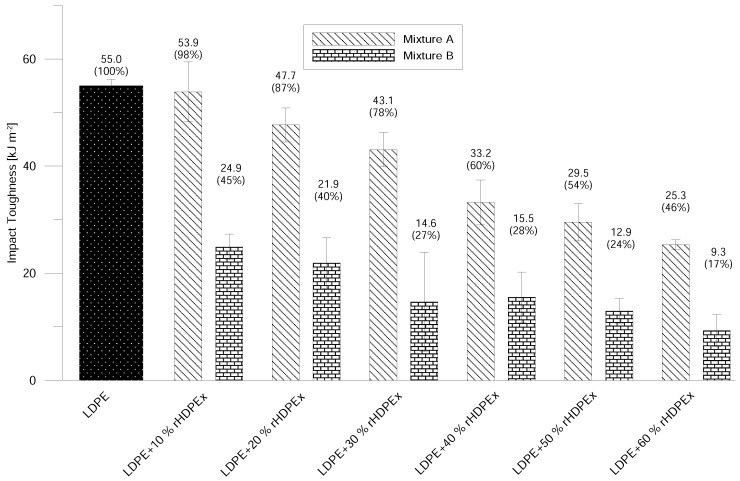
Impact toughness—Mixtures A and B.

The impact properties of Mixtures A and B are strongly influenced by the concentration of the rHDPEx filler. By the addition of 10% of filler in Mixture A, the changes in Charpy Notched Impact Strength are negligible, but further increases led to considerable decreases in the impact properties. The difference in the impact strength of virgin LDPE and a composite with the addition of 60 wt. % rHDPEx grit is 54%. The addition of rHDPEx filler in the form of a powder (Mixture B) causes significant changes in impact toughness. This is already detectable from the smallest content of the powdered filler. At the rHDPEx powder concentration of 10%, the impact toughness dropped by up to 45% of the value of the virgin LDPE. With further increases in filler content, impact toughness continually decreased, down to 17% that of the virgin LDPE (Figure 12). From the measured values, it follows that the Impact properties of both mixtures are affected by the filler content and the size of the filler particles.

**Figure 13:**
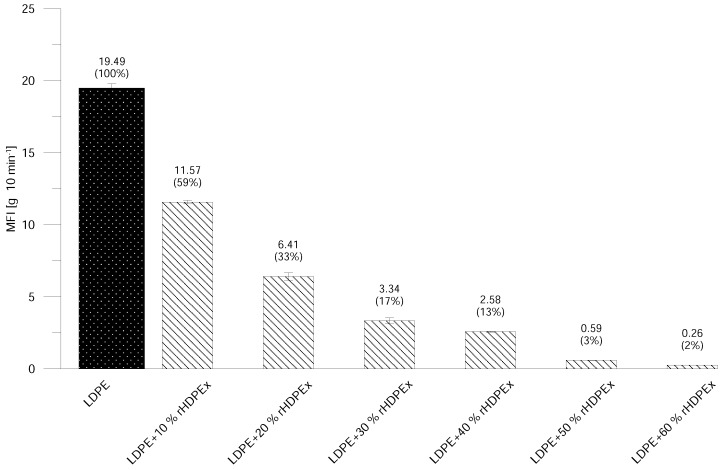
MFI—Mixture B.

**Figure 14:**
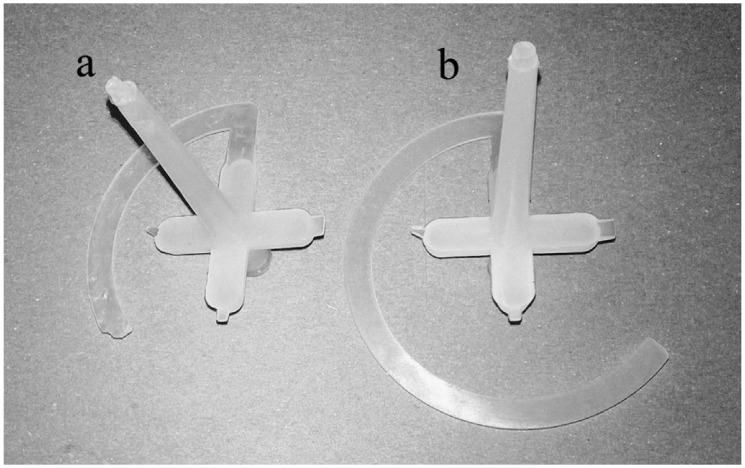
Resulting spirals: (**a**) LDPE + 60% rHDPEx (Mixtures A); and (**b**) LDPE.

**Figure 15:**
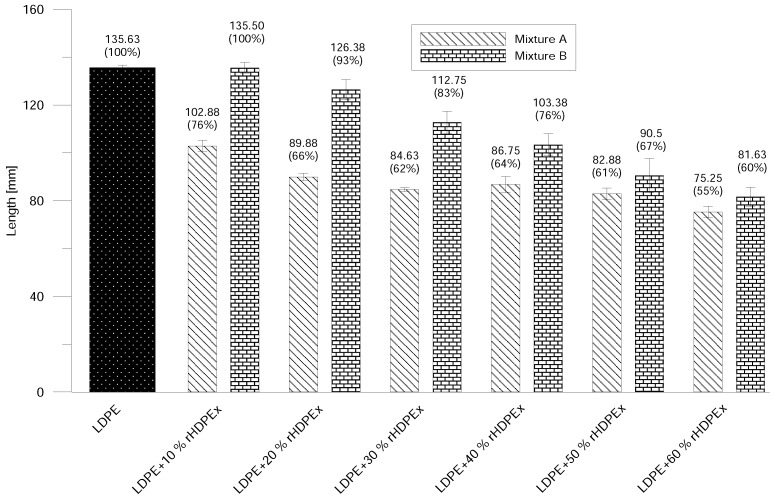
Polymer fluidity comparison—Mixtures A and B.

The basic processing parameter is represented by the MFI, which is a standard polymer flow behaviour test. Due to the crushed particles’ dimensions—and the impossibility of melting rHDPEx—it was impossible to make MFI measurements for Mixture A (LDPE + rHDPEx grit), so measurement was performed only for Mixture B. With an increasing dose of rHDPEx, the viscosity of Mixture B melts, with a MFI decrease to a minimum at a filler content of 60 wt. % (Figure 13). The melt flow gradually decreases in line with increases in the rHDPEx content. A Modified Spiral Test was performed to assess the melt flow of both mixtures. An injection mould was used (Figure 3). The spirals resulting from this test are depicted in Figure 14. This spiral fluidity test most closely simulates the real processing conditions. A huge drop in the mixture melt fluidity can be seen in Figure 15, when comparing virgin LDPE and LDPE with 60% of recycled irradiated HDPE (rHDPEx). The viscosity of this composite increases with the filler content, and there is no possibility to add more than 60% of filler (rHDPEx) to be processed. Already addition 10 wt. % of rHDPEx (Mixture A) causes drop of length of composite melt flow of 24% with subsequent slow decrease of fluidity with the filler content up to 45%, in the case of the highest filler content. In the case of Mixture B, 10 wt. % filler content causes no change of fluidity. Despite the relatively large difference in the length of melting of Mixtures A and B at low filler concentrations (10–30 wt. %), the final length of the spiral (as measured at the maximum concentration of rHDPEx), is comparable.

Although the MFI results and the Modified Spiral Test cannot be fully compared, both tests showed that the content and particle size of the filler significantly affect the flow properties of the LDPE + rHDPEx mixtures.

**Figure 16:**
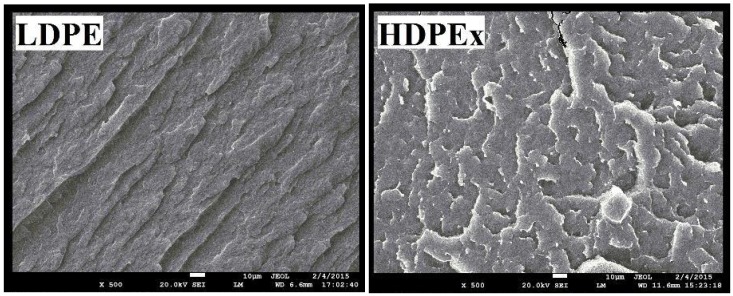
SEM—LDPE and HDPEx structure (500×).

**Figure 17:**
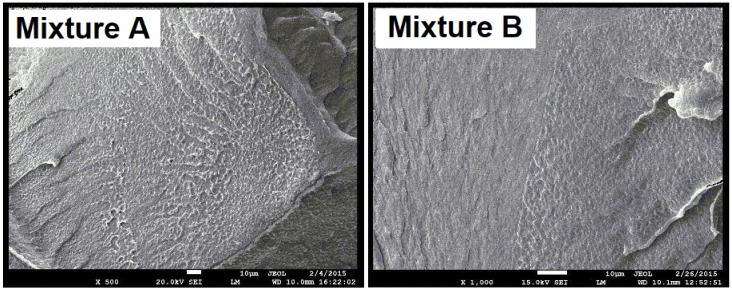
SEM—fracture surface structure of Mixtures A and B.

To be able to evaluate the structure of the mixtures, Microtome Cuts method and SEM Fracture Microscopy (Figure 16) were used to show the characteristic fracture structures of the LDPE and HDPEx mixtures. For the structure of both tested mixtures (Figure 17), large domains of rHDPEx in the LDPE matrix are characterised by very good adhesion characteristics of the two components, without any evidence of their separation. This tendency is very clearly observed when using the microtome cuts method, where the distinct interfaces between the matrix and the filler particles are clearly visible.

**Figure 18:**
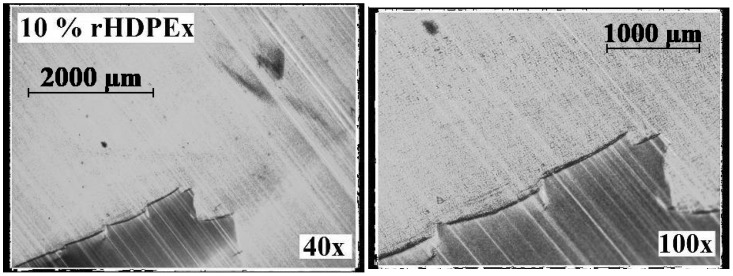
Microtome cuts of Mixture A.

**Figure 19:**
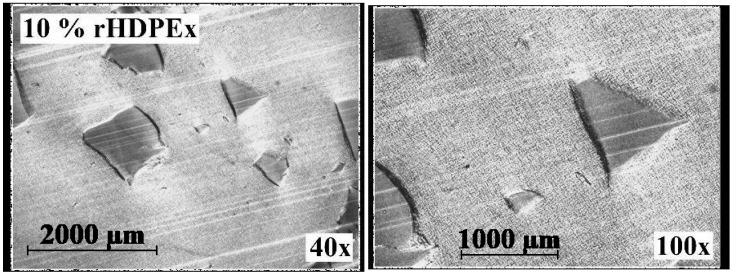
Microtome cuts of Mixture B.

It is visible from the structure of the mixtures, as shown in Figure 18 and Figure 19, that the adhesion between the matrix (LDPE) and the filler is very good and strong. The visible interface between the LDPE matrix and rHDPEx particles attests to the fact that the rHDPEx has a similar behaviour to filler. These findings correspond very well to the measured mechanical properties values. It is possible to say that irradiation of HDPE might also help to strengthen adhesion and overcome the problematic miscibility of HDPE and LDPE. Generally speaking, the basic purpose of the filling is to increase the bulk of the polymer and to improve its desired properties, at low cost. The utilization of recycled irradiated material as filler (in our study, rHDPEx), is a very good idea, and a successful step. Further research work being realized at this time shows that the principle discussed in this article could also be successfully used for processing other irradiated polymers that represent a big problem today. The principle itself is very simple, and the results described in this paper could bring added-value in the processability of this waste into a material with improved properties—mainly its mechanical properties. Through the appropriate blending of the mixtures, it is possible to modify the properties of the matrix (LDPE) to a relatively wide extent, to suit specific requirements (so-called “tailor-made” materials).

Results presented in this article show that there is potential to use rHDPEx as a filler up to 60 wt. %. Mechanical properties of the mixtures are influenced by the content of the fillers: use of rHDPEx brings improvement of E-moduli, Tensile strength, shore D, and micro-hardness to Mixtures A and B when compared with virgin LDPE, while the impact toughness, strain and fluidity all show decreases. Flow properties are also influenced by the size of rHDPEx particles. In Figure 15, it follows that even 10 wt. % of grit content in Mixture A causes a drop in fluidity of about 24% (and 34% with a filler content of 20 wt. %), while in Mixture B 10 wt. % of powdered rHDPEx brings no changes in fluidity and a content of 20 wt. %, causes a decrease of only 7%. The mechanical and flow properties of the tested mixtures are affected—not only by the filler content, but also has a significant effect on the size of filler particles, which depends on the ratio of the contact areas of the filler particles and their volume.

## 4. Conclusions

The aim of this paper was to show the results obtained from the utilization of recycled irradiated High Density PolyEthylene (rHDPEx). Underfloor heating pipe waste, made from HDPEx, was used for this study. The idea of using this material as a filler was studied with the aim of investigating the influence of the filler on the mechanical properties and processability of the mixture. The results show that this technology, in few steps—such as cleaning (if necessary), crushing/grinding of the product from irradiated polymer, and the addition of grit or powder as a filler to the origin polymer matrix, in the concentration up to 60 wt. %—could be very successfully implemented in the waste-processing field. The utilization of crushed/ground rHDPEx as a filler for virgin LDPE matrix seems to be the easiest—and most efficient way—to perform this form of waste processing. It is also possible to take further steps—e.g., the re-granulation of the prepared mixture—but this leads to a rise in production costs. This research paper offers answers to the problem of the re-utilization of irradiated high density polyethylene. Other materials are under research today, with the aim of making a general statement on the utilization of products made from irradiated polymers. The idea presented herein is very simple, and could be, without need for huge investments, used in practice to transform today’s unused waste into the required raw material for the production of a huge amount of useful products.

## Figures and Tables

**Table 1:** Injection moulding processing parameters.

**ARBURG ALLROUNDER 470H**		
Injection Velocity	60	mm s${}^{-1}$
Injection Pressure	45 (55 ${}^{1}$)	MPa
Cooling Time	30	s
Mould Temperature	40	${}^{\circ }$C
Holding Time	10	s
Holding Pressure	40 (55 ${}^{1}$)	MPa
**Temperature of Plasticizing Unit Zones**		
Temperature under the Hopper	40	${}^{\circ }$C
Temperature Zone 1	135	${}^{\circ }$C
Temperature Zone 2	140	${}^{\circ }$C
Temperature Zone 3	150	${}^{\circ }$C
Temperature Zone 4	160	${}^{\circ }$C
Temperature Zone 5	180	${}^{\circ }$C

${}^{1}$ used at the highest concentration of filler (60 wt. %).
